# Predicted Global Redistribution of Pyrophilous Beetle *Melanophila acuminata* (Coleoptera: Buprestidae) Under Climate Change

**DOI:** 10.1002/ece3.74317

**Published:** 2026-09-08

**Authors:** Jieqiong Wang, Shiqi Liu, Yingying Li, Aimin Shi, Yunchun Li, Ding Yang, Zhonghua Wei

**Affiliations:** ^1^ College of Life Sciences China West Normal University Nanchong Sichuan China; ^2^ College of Plant Protection China Agricultural University Beijing China; ^3^ State Key Laboratory of Green Pesticides Guizhou University Guiyang Guizhou China

**Keywords:** climate change, MaxEnt, *Melanophila acuminata*, pyrophilous beetle, suitable habitats

## Abstract

The pyrophilous beetle *Melanophila acuminata* is a decomposer of freshly burned wood, playing a critical role in nutrient cycling within forest ecosystems. To investigate the impact of climate change on the global distribution of *M. acuminata* , this study employed MaxEnt modeling in conjunction with 22 environmental variables under current and future conditions (2050s and 2070s) to conduct a comparative analysis of its suitable habitats. After rational screening, a total of 795 global occurrence records and 10 selected key environmental variables were incorporated into the MaxEnt model, which demonstrated excellent predictive accuracy (AUC ≥ 0.919). The results indicate that the mean temperature of the coldest quarter (Bio11) and the precipitation of the driest month (Bio14) are the primary factors limiting the distribution of *M. acuminata* . Under current climate conditions, its high‐suitability areas are concentrated in Northern Europe and the border region between the United States and Canada. Under the high‐emission scenario (SSP5‐8.5) in the 2070s, the high‐suitability area is projected to contract by nearly 36%, with the centroid shifting northwestward to higher elevation and an increase in habitat fragmentation. This projected contraction and fragmentation of suitable habitats may pose particular challenges for *M. acuminata* , given its known reliance on habitat connectivity for locating fire sites via its specialized infrared sensory system (Schmitz and Bousack 2012). However, direct validation of this mechanistic link requires further empirical investigation. Based on the predicted suitable habitats of *M. acuminata* in 2050s and 2070s, future conservation efforts should focus on high‐suitability areas in Northern Europe and Canada.

## Graphical abstract

*Melanophila acuminata* 's core suitable habitat is projected to shrink by ~36% and shift northwestward under high‐emission scenarios, with Northern Europe and Canada as key future conservation priorities.

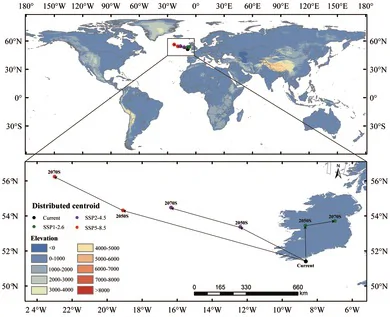

## 1 Introduction

Global warming is one of the major scientific challenges facing this century. Climate change is contributing to the decline of biodiversity and altering species distribution patterns (Bellard et al. 2012; Pecl et al. 2017; Chen et al. 2023), which can lead to changes in the structure and function of ecosystems (Bellard et al. 2012). Therefore, studying the geographical distribution patterns of species under climate change will provide data support for biodiversity conservation, resource management, and pest control. To better explore the relationship between climate change and species niches, various species distribution models have been developed, including maximum entropy modeling (MaxEnt) (Phillips et al. 2006), CLIMEX (Sutherst et al. 2004), Enphylo (Mondanaro et al. 2023), Genetic Algorithm for Rule Set Production (GARP) (Stockwell 1999), and the bioclimate analysis and prediction system (BIOCLIM) (Booth et al. 2014), among others. Among these models, the MaxEnt model is widely used due to its high accuracy, ease of use, and the relatively small number of species occurrence points required (Elith et al. 2006; Merow et al. 2013; Phillips et al. 2017).

According to existing research results, the effects of climate change on the distribution ranges of different insects are inconsistent: under future climatic conditions, some species are expected to expand their distribution ranges (Fu et al. 2024; Zhao et al. 2024; Aidoo et al. 2022), while others are predicted to shrink (Yu and Li 2024; Poloni et al. 2022). Therefore, predicting suitable habitats for species that hold resource value, economic value, and ecological value will benefit their conservation, management, and control.

The family Buprestidae is one of the larger groups within the order Coleoptera, comprising over 15,000 species (Bellamy 2008). Within this family, some species are collected or used as decorative items due to their vibrant colors, while others are considered pests in agriculture and forestry, such as *Agrilus mali* Matsumura, 1924 (Zhang et al. 2020), *Agrilus planipennis* Fairmaire, 1888 (Herms and McCullough 2014), and *Trachypteris picta* (Pallas, 1773) (Liu et al. 2025). Among buprestid species, *Melanophila acuminata* (DeGeer, 1774) is one of the well‐known pyrophilous beetles, with its distribution mainly confined to the Northern Hemisphere, including North America, Europe, and Asia (Bellamy 2008). It exhibits a strong preference for freshly burned trees (Apel 1988; Ricksecker 1885; VanDyke 1928). It is among the first decomposers to colonize fire‐affected forests after a large fire, laying its eggs under the bark of charred trees, where its larvae develop within the wood (Nicholson 1919). Consequently, it plays a crucial role in the decomposition of fire‐damaged and lightning‐struck wood. This species is capable of detecting wildfires from long distances, and the microscopic structure and sensitivity of its infrared receptors have been thoroughly investigated (Evans 1964; Schmitz and Bousack 2012).

To investigate the impact of climate change on the suitable habitats of *M. acuminata* , this study employed the MaxEnt model, utilizing 22 environmental factors (19 bioclimatic factors, global land cover type, global vegetation, and elevation) to explore changes in its potential distribution. The results will provide fundamental data for the monitoring and conservation of the pyrophilous beetle *M. acuminata* .

## 2 Materials and Methods

### 2.1 Obtaining and Processing Occurrence Data

**FIGURE 1:**
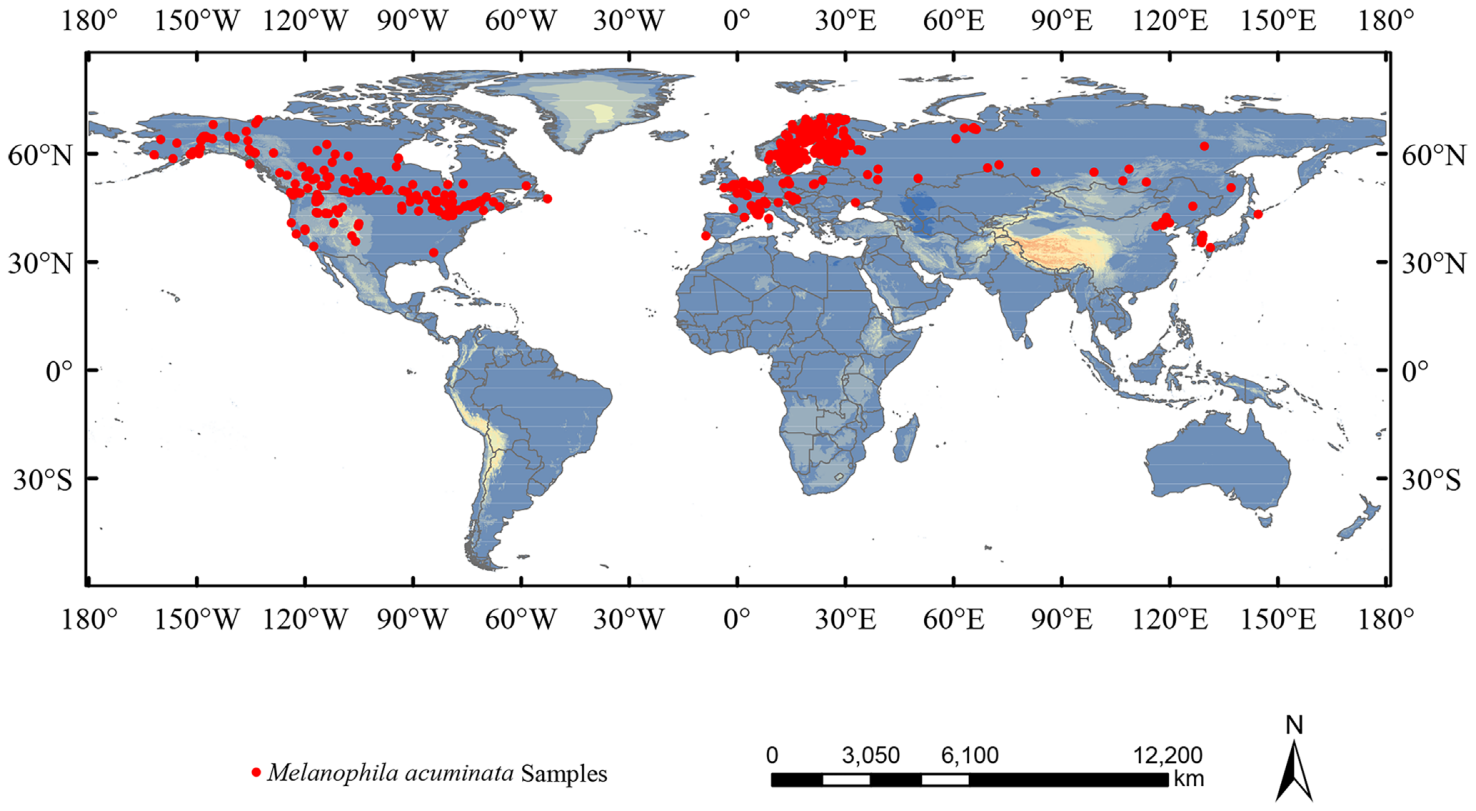
Global occurrence records of *Melanophila acuminata* . The gradient colors of the base map represent elevation. Red dots indicate georeferenced occurrence records of *Melanophila acuminata* .

Global distribution records of *M. acuminata* were primarily sourced from the Global Biodiversity Information Facility (GBIF, https://www.gbif.org/), the China National Knowledge Infrastructure (CNKI, https://www.cnki.net/) and the National Animal Collection Resource Center (NACR, http://museum.ioz.ac.cn/). To mitigate potential sampling bias, spatial autocorrelation, and the effects of environmental heterogeneity across the study area, we conducted thorough data cleaning and spatial filtering (Boria et al. 2014). Duplicate records with identical coordinates and collection dates were removed, while records with ambiguous geographic locations, questionable specimen information, or precision limited to state or provincial levels were excluded, as these factors can affect the accuracy of SDMs (Koo et al. 2025). Spatial thinning was then performed using ENMTools at a grid resolution of 2.5 arc minutes, retaining only one occurrence point per grid cell globally (Warren et al. 2021). A total of 1457 raw global occurrence points were initially collected (Figure 1). After data filtering, 795 valid occurrence points were retained. All coordinates were compiled into an Excel spreadsheet and converted to CSV format for subsequent construction and analysis of the MaxEnt ecological niche model on a global scale.

### 2.2 Environmental Variables

This study constructed an initial set of environmental variables comprising 19 bioclimatic variables with a spatial resolution of 2.5 arc minutes, which serve as the core climatic factors. In addition, three categories of non‐climatic environmental variables were included: land cover type (gm_lc), percentage of vegetation cover (gm_ve), and elevation (elev). These variables collectively characterize the climatic and geographic conditions influencing the distribution of *M. acuminata* . The 19 bioclimatic variables were obtained from the WorldClim database (http://www.worldclim.org/), while data on land cover type and percentage of vegetation cover were sourced from the Global Land Cover Database (https://globalmaps.github.io/), with vegetation cover values ranging from 0% to 100% (Kobayashi et al. 2017). Elevation data were derived from the EarthEnv database (https://www.earthenv.org/).

To mitigate the risk of model overfitting and reduced predictive reliability caused by multicollinearity among environmental variables, we employed Pearson correlation analysis to screen the initial set of environmental variables (Warren et al. 2021) (Figure S1; Table S1). Pearson correlation coefficients were calculated for all pairs of variables, using an absolute correlation coefficient threshold of |r| > 0.85 to identify strong multicollinearity (Wei et al. 2018; Zhong et al. 2023). For variable groups exhibiting strong multicollinearity, redundant variables were eliminated based on the ecological habits of *M. acuminata* and the ecological significance of each predictor. As a thermophilic and xylophagous insect, the survival, development, and dispersal activity of *M. acuminata* are mainly restricted by thermal conditions. Therefore, we prioritized temperature‐related bioclimatic variables with direct biological implications, followed by precipitation‐related variables. Variables that only indirectly affect insect populations were excluded to avoid information redundancy, retaining only independent core environmental variables with clear ecological interpretability for the subsequent construction of the MaxEnt model. Finally, 10 selected environmental variables (Table 1) were used as input factors for the model. The Jackknife test was performed to evaluate the relative importance of each variable in determining species distribution (Warren et al. 2021).

**TABLE 1:** The selected 10 environmental variables used in the MaxEnt model.

Bioclimatic variable	Description	Unit	Spatial resolution	Data source	Contribution	Permutation importance
Bio01	Annual mean temperature	°C	2.5 arc‐min	WorldClim v2.1	10	2.5
Bio03	Isothermality	—	2.5 arc‐min	WorldClim v2.1	4.1	9.2
Bio05	Maximum temperature of the warmest month	°C	2.5 arc‐min	WorldClim v2.1	2.8	24.2
Bio08	Mean temperature of the wettest quarter	°C	2.5 arc‐min	WorldClim v2.1	2.0	6.2
Bio10	Mean temperature of the warmest quarter	°C	2.5 arc‐min	WorldClim v2.1	0.9	11.2
Bio11	Mean temperature of the coldest quarter	°C	2.5 arc‐min	WorldClim v2.1	19.2	30.5
Bio14	Precipitation of the driest month	mm	2.5 arc‐min	WorldClim v2.1	39.7	4.3
gm_lc	Global land cover type	Classified category	2.5 arc‐min	Global Land Cover database	10.1	3.1
gm_ve	Global vegetation (percent tree cover)	%	2.5 arc‐min	Global Maps database	8.8	0.4
elev	Elevation	m	2.5 arc‐min	EarthEnv	2.4	8.5

### 2.3 Modeling Methods

This study employed MaxEnt v3.4.4 and ArcGIS v10.2 to develop a global habitat suitability model for *M. acuminata* . To avoid overfitting, we performed parameter optimization for feature classes and regularization multiplier using the Kuenm R package. The final optimized settings were feature classes: LQPH (Linear, Quadratic, Product, Hinge), regularization multiplier: 1. The Jackknife test was performed to evaluate the relative importance of each variable in determining species distribution (Warren et al. 2021). Model parameters were configured as follows: the maximum iteration number was set to 500, and the number of background points was fixed at 10,000 (Zhu and Jiao 2016; Phillips et al. 2006). Background points were randomly generated within the species' accessible range to alleviate spatial autocorrelation, and spatial thinning of occurrence records was implemented via ENMTools (2.5 arc‐minute grid, one record per cell) to mitigate sampling bias. All species occurrence records were randomly partitioned into training and testing datasets at a 75 to 25 ratio. The Bootstrap approach with 10 replicate runs was adopted to minimize stochastic noise, reduce model randomness, and generate averaged prediction outputs.

Model performance was evaluated using the receiver operating characteristic (ROC) curve, with the area under the curve (AUC) serving as the metric for predictive accuracy. AUC values range from 0 to 1, where an AUC ≥ 0.8 indicates good predictive performance and AUC ≥ 0.9 indicates excellent performance (Phillips et al. 2006; Phillips and Dudík 2008). Additionally, model complexity and goodness‐of‐fit were comprehensively assessed using the omission rate and the corrected Akaike Information Criterion (AICc) values (Anderson et al. 2003; Warren and Seifert 2011). For the final optimized MaxEnt model, the omission rate at the 5% threshold was 0.077 and the AICc value was 3049.88.

Current climate data from the 1970–2000 period served as the baseline for comparison. Future climate scenarios were derived from the BCC‐CSM2‐MR global climate model under the Coupled Model Intercomparison Project Phase 6 (CMIP6) (Eyring et al. 2016; Riahi 2017). Three Shared Socioeconomic Pathway emission scenarios were selected: SSP1‐2.6 (low emission), SSP2‐4.5 (medium emission), and SSP5‐8.5 (high emission), covering the periods of 2041–2060 (2050s) and 2061–2080 (2070s).

In ArcGIS v10.2, suitable habitats were classified using the Maximum Training Sensitivity Plus Specificity (MTSPS) threshold in conjunction with the Jenks natural breaks method (Liu et al. 2016). Based on the presence probability output from the model, the study area was categorized into four classes: unsuitable habitat, low‐suitable habitat, moderately suitable habitat, and highly suitable habitat. Area statistics for each category were compiled using the SDM Toolbox v2.4.

## 3 Results

### 3.1 Evaluation of Model Prediction Accuracy

The AUC values for both current and future climate scenarios exceed 0.9 (Figure S2), indicating that the model demonstrates strong discriminative ability. Generally, an AUC value closer to 1 reflects better predictive performance, with a value of 1 indicating a perfect correspondence between the predicted and actual distribution of the species (Wang et al. 2007). In this study, the MaxEnt model for *M. acuminata* achieved excellent predictive performance across all climate scenarios (AUC ≥ 0.919). These results demonstrate the high reliability of the model and confirm its capacity to provide accurate predictions of the potential distribution of this species under both current and future climate conditions.

### 3.2 Potential Suitable Distribution Areas Under Current Climate Scenarios

**FIGURE 2:**
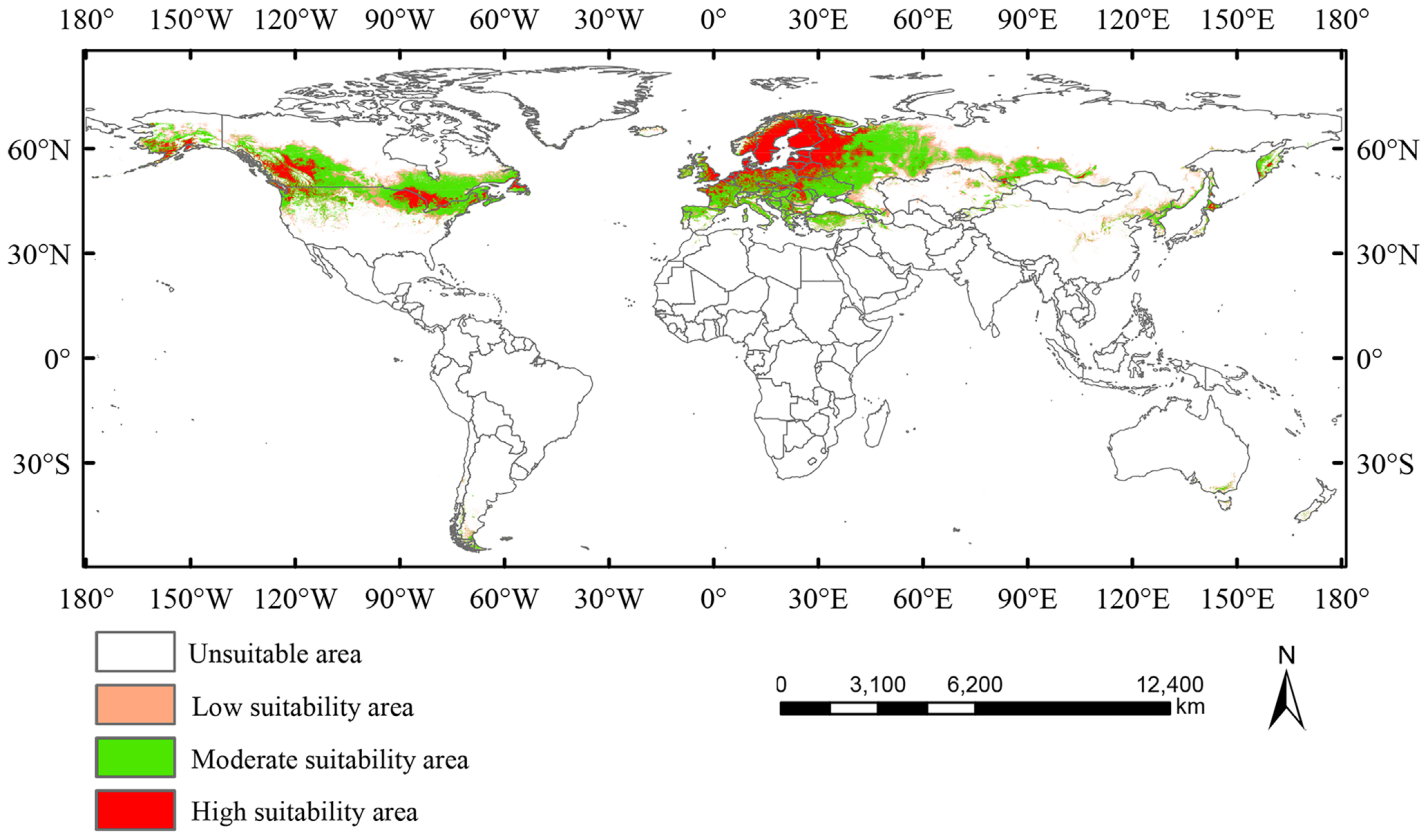
The suitable areas of *Melanophila acuminata* under current climate conditions.

Based on the filtered environmental variables and the MaxEnt model, the global potential distribution of *M. acuminata* under current climate scenarios was predicted, with a particular emphasis on mapping and analysis in East Asia (Figure 2). The simulation results indicated that under current climatic conditions, suitable habitats of *M. acuminata* are primarily distributed in a typical disjunct pattern on a global scale. High‐suitability areas are concentrated in northeastern North America (the border region between Canada and the United States) and northern Europe. Moderately suitable areas form a continuous belt spanning the cold‐temperate to temperate zones across North America, Europe, and North Asia. Low‐suitability areas are distributed in the transitional zones surrounding the high‐suitability areas, with scattered and isolated suitable patches occurring in southern South America, southeastern Australia, and New Zealand.

Area statistics showed that under the current climate scenario, highly suitable, moderately suitable, and low‐suitability habitats in the study area accounted for 4.08%, 9.16%, and 3.49% of the total land area, respectively (Table S2). Model evaluation indicated that the mean AUC value from the replicate runs reached 0.944, with a low standard deviation (Figure S2), suggesting that the model predictions for the potential habitat of *M. acuminata* are highly reliable and effectively reflect the distribution characteristics of this species within the study area.

**FIGURE 3:**
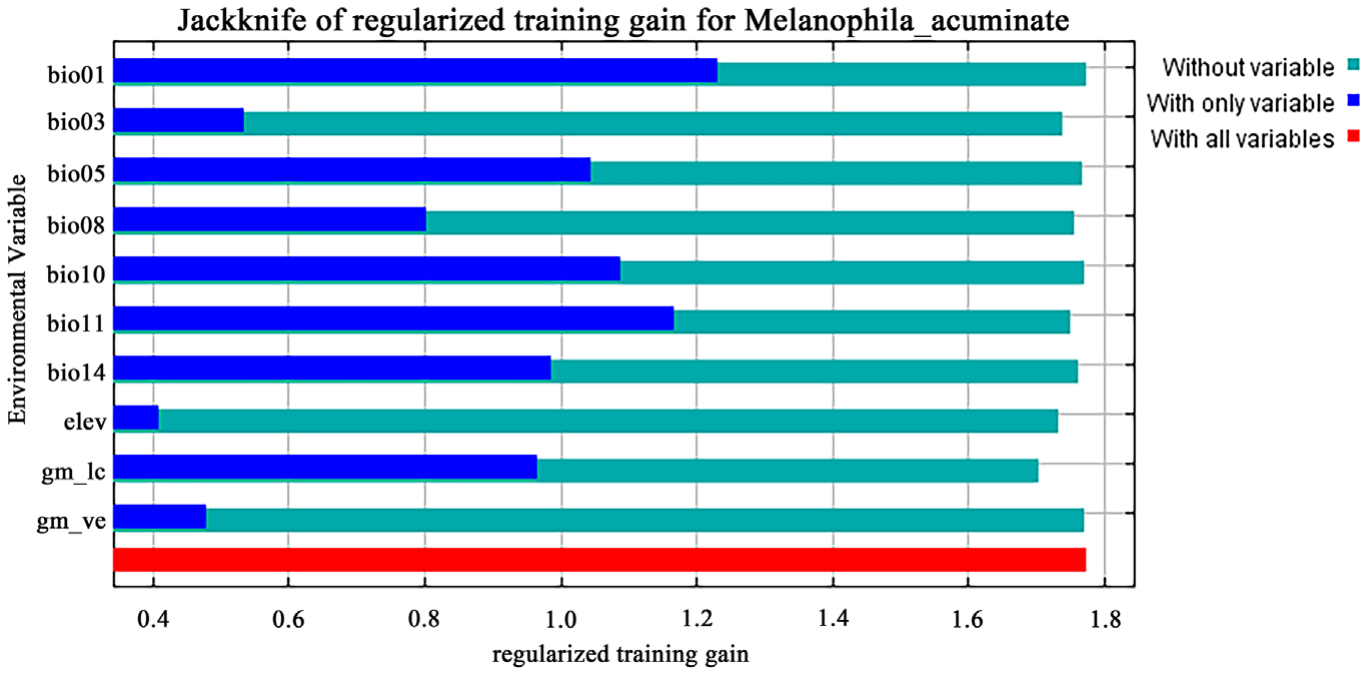
Jackknife test of variable importance in the *Melanophila acuminata* suitability distribution.

According to the Jackknife test results for variable importance (Figure 3), bio01 exhibited the highest regularized training gain in the with only variable scenario, establishing it as the primary environmental factor driving the distribution of *M. acuminata* . In contrast, factors elev and gm_ve demonstrated the lowest gains in the with only variable scenario, indicating their limited ability to independently explain species distribution. In terms of variable uniqueness, the removal of gm_lc resulted in the lowest model gain in the without variable scenario, suggesting that this variable contains unique information not captured by other factors. Following closely were elev and bio03, whose removal also led to substantial declines in model gain, highlighting their significant independent contributions.

**FIGURE 4:**
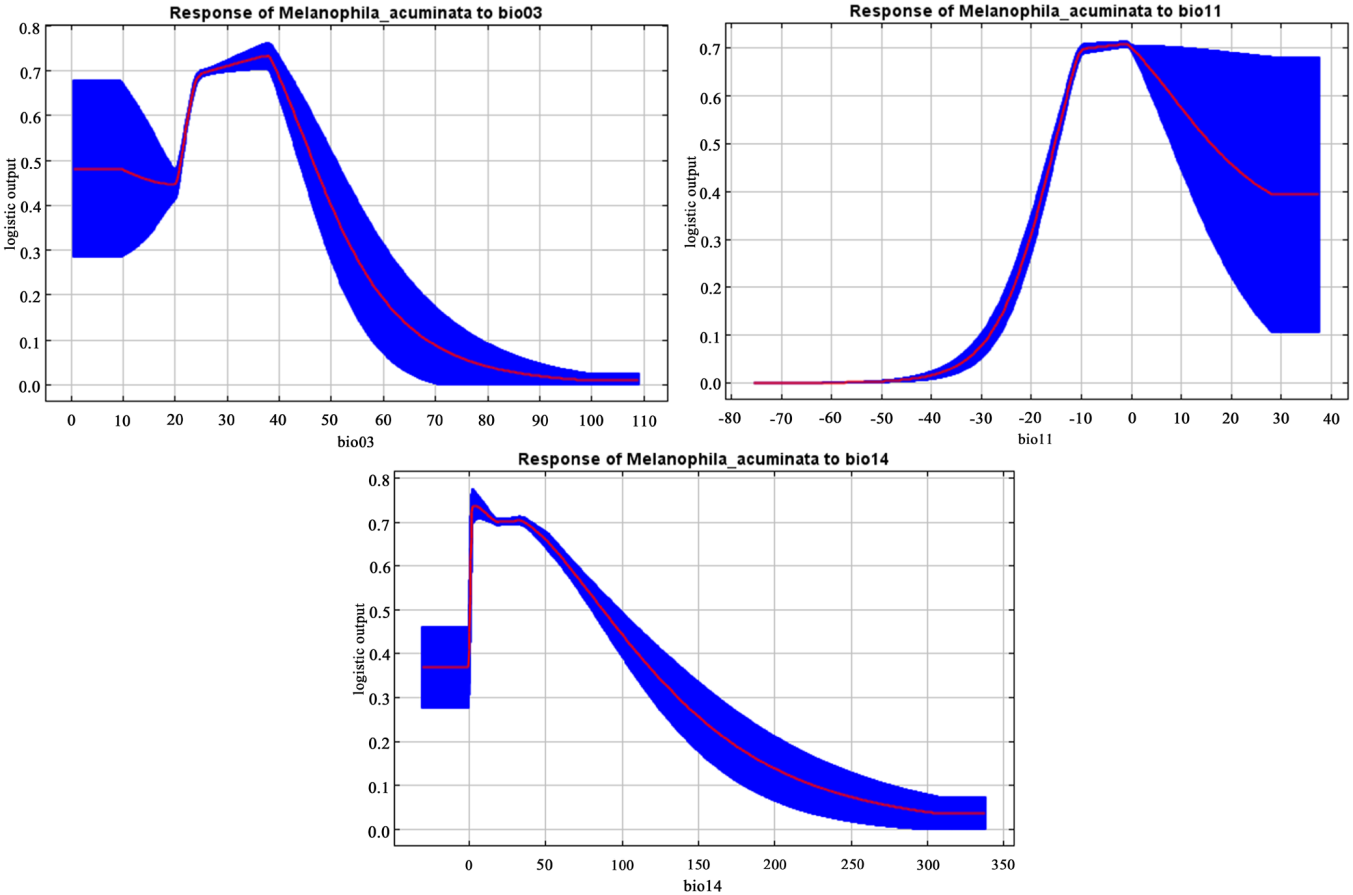
Response curves of the three major environmental variables important for *Melanophila acuminata* .

The response curves of the dominant environmental variables for *M. acuminata* were generated using the MaxEnt model (Figure 4). The results indicate that, in terms of suitable habitat, the optimal range for bio03 is approximately 21–41, with an optimum value of around 37, reflecting a preference for moderate temperature stability. For bio11, the suitable range is approximately −15°C to 15°C, with an optimum value of about −1°C. Regarding bio14, the suitable range is approximately 0–87 mm, with an optimum value of about 3 mm, underscoring that extremely dry moisture conditions are a critical requirement for its habitat.

### 3.3 Change of Suitable Areas Under Future Climate Conditions

**FIGURE 5:**
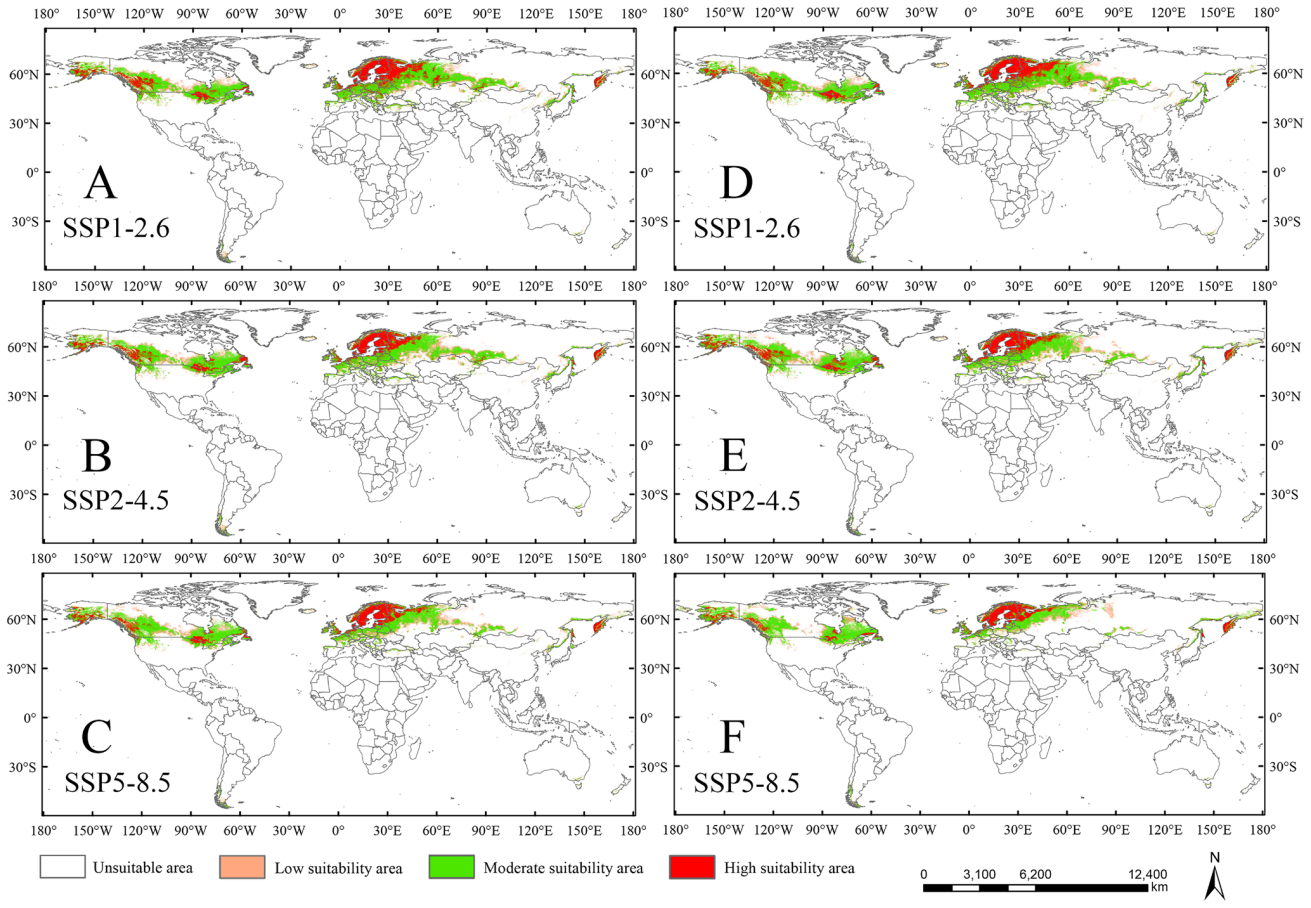
Predicted suitable areas for *Melanophila acuminata* under three climate scenarios (SSP1‐2.6, SSP2‐4.5, SSP5‐8.5) in the 2050s (A–C) and 2070s (D–F), respectively.

In future climate scenarios (Figure 5), the suitable habitat for *M. acuminata* is projected to contract overall and migrate toward higher latitudes and elevations. Quantitative changes in habitat gain, habitat loss, and stable suitable area across scenarios are summarized in Table 2. Under the SSP1‐2.6 scenario in the 2050s, suitable habitats are primarily distributed in eastern China, the Korean Peninsula, and Japan, exhibiting a discontinuous distribution pattern. As greenhouse gas emission pathways shift toward the higher‐emission SSP2‐4.5 and SSP5‐8.5 scenarios, the spatial extent of highly suitable habitats gradually declines, accompanied by substantial losses of moderately suitable habitats across southern China and Southeast Asia. Among the three scenarios for 2041–2060, SSP5‐8.5 yields the largest habitat‐loss area (6,327,658.37 km^2^, 4.68%), while habitat gain remains limited under all scenarios. By the 2070s, the distribution of suitable habitats under the SSP1‐2.6 scenario remains relatively stable compared to the 2050s. However, under the SSP2‐4.5 scenario, suitable habitats further contract toward the northeast, while under the SSP5‐8.5 scenario, the range of suitable habitats decreases dramatically, with only sparse distributions remaining in southern Japan and the southeastern coastal regions of China.

**TABLE 2:** Proportion (%) and area (km^2^) of habitats at different suitability levels for *Melanophila acuminata* under current and future CMIP6‐SSP climate scenarios.

	Unsuitable area (%)	Low suitability area (%)	Moderate suitability area (%)	High suitability area (%)	Unsuitable area (km^2^)	Low suitability area (km^2^)	Moderate suitability area (km^2^)	High suitability area (km^2^)
Current	83.28	3.49	9.16	4.08	112,690,993.59	4,727,648.85	12,389,269.97	5,514,704.84
2041–2060, SSP1‐2.6	83.71	3.18	8.97	4.13	113,278,128.12	4,307,736.53	12,142,552.35	5,594,200.24
2041–2060, SSP2‐4.5	84.67	3.33	8.59	3.40	114,584,065.25	4,505,637.63	11,628,113.03	4,604,801.34
2041–2060, SSP5‐8.5	85.78	3.28	7.86	3.07	116,079,910.08	4,443,776.41	10,639,932.17	4,158,998.59
2061–2080, SSP1‐2.6	84.02	3.21	8.90	3.87	113,701,183.96	4,340,593.17	12,042,398.99	5,238,441.12
2061–2080, SSP2‐4.5	84.78	3.29	8.43	3.50	114,720,546.66	4,447,445.76	114,13,083.25	4,741,541.59
2061–2080, SSP5‐8.5	87.61	2.98	6.80	2.62	118,553,217.03	4,028,500.80	9,202,187.85	3,538,711.57

Overall, as the intensity of greenhouse gas emissions increases (SSP‐2.6 to SSP5‐8.5) and the projection period extends (2041–2060 to 2061–2080), the areas of high and medium suitability habitats continue to shrink, resulting in a more fragmented spatial distribution. The range of low‐suitability habitats shows slight fluctuations, while the proportion of unsuitable areas significantly expands. Compared to the SSP1‐2.6 and SSP2‐4.5 scenarios, the retreat of suitable habitats under the high‐emission scenario of SSP5‐8.5 is more pronounced, leading to an overall decline in habitat suitability. The area of stable suitable habitat gradually decreases with rising emission intensity (Table 2). This trend indicates that future climate warming will persistently restrict the potential distribution range of *M. acuminata* , putting its suitable habitats at significant risk of loss.

### 3.4 Centroid Migration Dynamics Under Future Climate Scenarios

**FIGURE 6:**
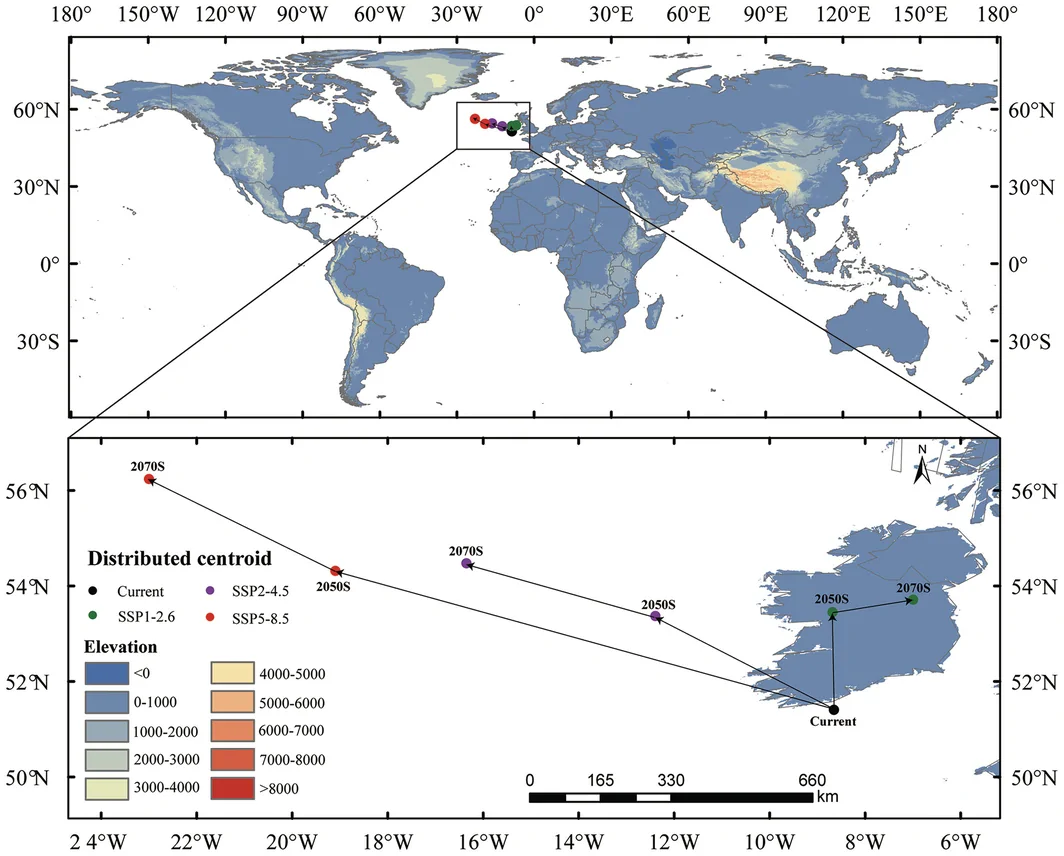
Centroid migration of *Melanophila acuminata* under different climate conditions.

To elucidate the spatial redistribution pattern under future climate change, this study analyzed the migration trajectories of the suitable area centroid of *M. acuminata* (Figure 6). Under current climate conditions, the centroid is located at (−8.65° E, 51.40° N). Future climate scenarios project a consistent northwestward shift in the centroid, with the magnitude of migration increasing as the intensity of emission pathways rises.

Overall, the centroid of suitable habitats for *M. acuminata* is projected to shift northwestward and to higher elevations under future climate scenarios, with the intensity of migration increasing in response to higher emission pathways. This indicates that the suitable habitats for this species will undergo continuous spatial adjustments as climate conditions change.

## 4 Discussion

Numerous studies have demonstrated that climatic factors often exert more significant effects on species distributions than other environmental variables at large spatial scales (Wei et al. 2020, 2018). In this study, the MaxEnt model was employed to predict the global potential suitable distribution of *M. acuminata* based on ten selected key environmental variables and 795 occurrence records from around the globe. Two future periods (the 2050s and 2070s) and three Shared Socioeconomic Pathways (SSP1‐2.6, SSP2‐4.5, and SSP5‐8.5) were selected to assess the impacts of climate change on the potential distribution patterns of this species. The optimized MaxEnt model effectively reduced model complexity and improved the fit between predicted results and actual distributions (Wang et al. 2025). Model evaluation indicated that predictions in this study were consistent with the known distribution of the species (Yu et al. 2017), and the AUC values under all climate scenarios were ≥ 0.919, demonstrating the high accuracy of our MaxEnt model in predicting suitable habitats for *M. acuminata* (Phillips et al. 2006; Janitza et al. 2013).

The results indicated that the response of suitable habitats for *M. acuminata* to climate change is closely linked to greenhouse gas emissions intensity. Under low‐emission scenarios, the area of highly suitable habitat remains largely stable or may expand slightly; in contrast, under medium‐ and high‐emission scenarios, the area of suitable habitat continues to shrink. By the 2070s, under the high‐emission scenario, the high‐suitability area is projected to decrease by nearly 36% compared with the current baseline, while unsuitable habitats are expected to become increasingly widespread, resulting in highly fragmented habitats. This pattern of change contrasts markedly with the general trend observed in most other buprestid beetles, which typically expand toward higher latitudes and elevations in response to climate warming.

Studies about the impact of climate change on the distribution of buprestid beetles remain limited (Zhang et al. 2024; Liu et al. 2025; Yin et al. 2026). Under projected future climate scenarios, suitable habitats for *Agrilus mali* and *Trachypteris picta* are anticipated to expand and migrate northward (Zhang et al. 2024; Liu et al. 2025), which is consistent with findings from studies on other insect species (Aidoo et al. 2022; Fu et al. 2024; Hong et al. 2024). The distribution of *Agrilus planipennis* is predominantly constrained by the presence of its host *Fraxinus* sp., rather than climatic factors (Sobek‐Swant et al. 2012). We identified that bio14 and bio11 are the two primary limiting factors for the suitable habitats of the pyrophilous beetle *M. acuminata* . These results align with the established consensus that host plants, water availability, and temperature are critical determinants of species distribution. In contrast to global land cover types and vegetation, elevation appears to exert minimal influence on the distribution of *M. acuminata* .

Notably, *M. acuminata* possesses highly specialized infrared receptors that enable it to detect infrared radiation from fires at distances ranging from tens to hundreds of kilometers (Schmitz and Bousack 2012). This ability allows the species to rapidly locate and colonize recently burned forests; however, it also makes *M. acuminata* highly sensitive to habitat connectivity. The fragmentation of suitable habitats driven by future climate warming is likely to severely impede effective population dispersal among different fire sites, thereby exacerbating the risk of local extinction. *M. acuminata* serves as a decomposer in forest ecosystems, recycling nutrients through the breakdown of organic debris and facilitating nutrient cycling (Barrios 2007). Its absence could potentially slow decomposition rates, alter soil nutrient dynamics, and diminish soil fertility; however, the extent of these impacts will depend on functional redundancy within the ecosystem.

It should be noted that our conservation suggestion is derived from climate‐based habitat suitability simulation. The model does not integrate fire regime and fine‐scale local habitat features, which are also vital for the survival of *M. acuminata* . Although northern Europe and Canada exhibit high climatic suitability for this species, its long‐term persistence also relies on appropriate fire events and intact habitat connectivity. Accordingly, this conservation priority represents a climate‐driven reference, and practical conservation implementation requires further combination with field investigations and local fire‐regime information.

Additionally, future habitat‐suitability projections in this study were generated solely from a single GCM (BCC‐CSM2‐MR), which may introduce model‐related uncertainty. Our projections should therefore be considered in light of this limitation, and multi‐GCM ensemble simulation is recommended for further research. Given the predicted high suitable habitats, future conservation efforts for *M. acuminata* are expected to focus primarily on northern Europe and Canada.

## 5 Conclusions

In this study, we used the MaxEnt model to project the distribution patterns and changing trends of suitable habitats for *M. acuminata* under current and future climate scenarios. The results suggest that the mean temperature of the coldest quarter and the precipitation of the driest month are the primary environmental factors influencing the distribution of *M. acuminata* . Under various future climate scenarios, the overall area of suitable habitat for *M. acuminata* is projected to gradually shrink, with the magnitude of this reduction closely correlated with the intensity of greenhouse gas emissions. Under high‐emission scenarios, the contraction of suitable habitats is most pronounced, leading to a substantial decrease in high‐suitability areas. As a typical pyrophilous beetle, *M. acuminata* is likely to be particularly sensitive to habitat changes. Habitat fragmentation associated with climate warming may impede its dispersal and population persistence, although direct evidence for such effects remains limited. Therefore, we recommend prioritizing the monitoring and conservation of highly suitable regions such as northern Europe and Canada, as these areas are predicted to sustain suitable habitats under future climate scenarios. The findings of this study provide a preliminary scientific basis for the conservation management and climate‐adapted protection strategies; however, further empirical studies are needed to validate these predictions.

## Author Contributions

**Jieqiong Wang:** data curation (equal), software (equal), validation (equal), visualization (equal), writing – original draft (equal). **Shiqi Liu:** validation (equal), visualization (equal). **Yingying Li:** validation (equal), visualization (equal). **Aimin Shi:** conceptualization (equal), funding acquisition (equal). **Yunchun Li:** conceptualization (equal), funding acquisition (equal). **Ding Yang:** conceptualization (equal), funding acquisition (equal), resources (equal), supervision (equal). **Zhonghua Wei:** formal analysis (equal), investigation (equal), methodology (equal), project administration (equal), resources (equal), supervision (equal), writing – original draft (equal), writing – review and editing (equal).

## Funding

This study was funded by the Natural Science Foundation of Sichuan Province (2025NSFSC2117), the National Natural Science Foundation of China (32130012, 32670609), the Natural Science Special (Special Post) Scientific Research Fund Project of Guizhou University (2023‐06), and Qiankehe Platform Talent (BQW [2024] 012).

## Conflicts of Interest

The authors declare no conflicts of interest.

## Supporting information


**Table S1:** Pearson correlation coefficient matrix of all initial environmental variables.
**Table S2:** Potential suitable area of *Melanophila acuminata* in China under different scenarios.
**Figure S1:** Pearson correlation analysis of environmental variables.
**Figure S2:** The ROC curve for predicting the potential distribution range of *Melanophila acuminata* based on the MaxEnt model.

## Data Availability

The data supporting this study are openly available in the Dryad Digital Repository at https://doi.org/10.5061/dryad.r4xgxd2v0 upon acceptance.
