# Supplementary material for: Predicted Global Redistribution of Pyrophilous Beetle Melanophila acuminata (Coleoptera: Buprestidae) Under Climate Change
Source: Ecol Evol. 2026 Sep 8;16(9):e74317. doi: 10.1002/ece3.74317 (PMC13553553; doi:10.1002/ece3.74317)
Supplement: Supplementary file 1 [file ECE3-16-e74317-s001.docx]

**Table S1.** Pearson correlation coefficient matrix of all initial environmental variables.

|  | bio01 | bio02 | bio03 | bio04 | bio05 | bio06 | bio07 | bio08 | bio09 | bio10 | bio11 | bio12 | bio13 | bio14 | bio15 | bio16 | bio17 | bio18 | bio19 | elev | gm_lc | gm_ve |
| --- | --- | --- | --- | --- | --- | --- | --- | --- | --- | --- | --- | --- | --- | --- | --- | --- | --- | --- | --- | --- | --- | --- |
| bio01 | 1.00 | –0.02 | 0.68 | –0.72 | 0.69 | 0.90 | –0.66 | –0.02 | 0.77 | 0.81 | 0.94 | 0.45 | 0.37 | 0.33 | –0.17 | 0.39 | 0.39 | 0.05 | 0.46 | –0.05 | 0.14 | –0.05 |
| bio02 | –0.02 | 1.00 | 0.36 | 0.30 | 0.51 | –0.26 | 0.51 | –0.11 | 0.01 | 0.20 | –0.16 | –0.19 | –0.09 | –0.28 | 0.27 | –0.09 | –0.25 | –0.15 | –0.11 | 0.59 | 0.10 | –0.15 |
| bio03 | 0.68 | 0.36 | 1.00 | –0.74 | 0.41 | 0.72 | –0.59 | –0.40 | 0.75 | 0.33 | 0.76 | 0.27 | 0.24 | 0.13 | –0.08 | 0.26 | 0.20 | –0.26 | 0.47 | 0.43 | 0.03 | –0.09 |
| bio04 | –0.72 | 0.30 | –0.74 | 1.00 | –0.08 | –0.93 | 0.97 | 0.35 | –0.78 | –0.18 | –0.91 | –0.42 | –0.31 | –0.36 | 0.31 | –0.33 | –0.39 | 0.10 | –0.53 | –0.14 | 0.09 | –0.03 |
| bio05 | 0.69 | 0.51 | 0.41 | –0.08 | 1.00 | 0.37 | 0.05 | 0.11 | 0.44 | 0.92 | 0.45 | 0.17 | 0.15 | 0.04 | 0.03 | 0.16 | 0.10 | –0.02 | 0.17 | 0.04 | 0.26 | –0.13 |
| bio06 | 0.90 | –0.26 | 0.72 | –0.93 | 0.37 | 1.00 | –0.91 | –0.20 | 0.83 | 0.51 | 0.99 | 0.45 | 0.34 | 0.37 | –0.29 | 0.36 | 0.42 | –0.06 | 0.53 | –0.02 | 0.01 | 0.00 |
| bio07 | –0.66 | 0.51 | –0.59 | 0.97 | 0.05 | –0.91 | 1.00 | 0.27 | –0.70 | –0.13 | –0.86 | –0.41 | –0.30 | –0.38 | 0.32 | –0.32 | –0.40 | 0.05 | –0.49 | 0.03 | 0.11 | –0.06 |
| bio08 | –0.02 | –0.11 | –0.40 | 0.35 | 0.11 | –0.20 | 0.27 | 1.00 | –0.50 | 0.27 | –0.18 | –0.17 | –0.06 | –0.17 | 0.20 | –0.10 | –0.18 | 0.46 | –0.49 | –0.38 | 0.05 | 0.09 |
| bio09 | 0.77 | 0.01 | 0.75 | –0.78 | 0.44 | 0.83 | –0.70 | –0.50 | 1.00 | 0.45 | 0.84 | 0.39 | 0.30 | 0.26 | –0.21 | 0.33 | 0.32 | –0.31 | 0.62 | 0.15 | 0.03 | –0.06 |
| bio10 | 0.81 | 0.20 | 0.33 | –0.18 | 0.92 | 0.51 | –0.13 | 0.27 | 0.45 | 1.00 | 0.57 | 0.28 | 0.25 | 0.15 | 0.01 | 0.26 | 0.21 | 0.13 | 0.20 | –0.20 | 0.27 | –0.09 |
| bio11 | 0.94 | –0.16 | 0.76 | –0.91 | 0.45 | 0.99 | –0.86 | –0.18 | 0.84 | 0.57 | 1.00 | 0.46 | 0.37 | 0.36 | –0.25 | 0.38 | 0.42 | –0.03 | 0.53 | 0.03 | 0.04 | –0.01 |
| bio12 | 0.45 | –0.19 | 0.27 | –0.42 | 0.17 | 0.45 | –0.41 | –0.17 | 0.39 | 0.28 | 0.46 | 1.00 | 0.84 | 0.79 | –0.27 | 0.89 | 0.83 | 0.49 | 0.84 | –0.07 | 0.07 | –0.01 |
| bio13 | 0.37 | –0.09 | 0.24 | –0.31 | 0.15 | 0.34 | –0.30 | –0.06 | 0.30 | 0.25 | 0.37 | 0.84 | 1.00 | 0.40 | 0.25 | 0.99 | 0.45 | 0.54 | 0.68 | –0.02 | 0.07 | 0.00 |
| bio14 | 0.33 | –0.28 | 0.13 | –0.36 | 0.04 | 0.37 | –0.38 | –0.17 | 0.26 | 0.15 | 0.36 | 0.79 | 0.40 | 1.00 | –0.68 | 0.46 | 0.98 | 0.40 | 0.62 | –0.11 | 0.05 | –0.01 |
| bio15 | –0.17 | 0.27 | –0.08 | 0.31 | 0.03 | –0.29 | 0.32 | 0.20 | –0.21 | 0.01 | –0.25 | –0.27 | 0.25 | –0.68 | 1.00 | 0.17 | –0.67 | 0.07 | –0.24 | 0.08 | 0.07 | –0.03 |
| bio16 | 0.39 | –0.09 | 0.26 | –0.33 | 0.16 | 0.36 | –0.32 | –0.10 | 0.33 | 0.26 | 0.38 | 0.89 | 0.99 | 0.46 | 0.17 | 1.00 | 0.51 | 0.52 | 0.74 | –0.03 | 0.08 | –0.01 |
| bio17 | 0.39 | –0.25 | 0.20 | –0.39 | 0.10 | 0.42 | –0.40 | –0.18 | 0.32 | 0.21 | 0.42 | 0.83 | 0.45 | 0.98 | –0.67 | 0.51 | 1.00 | 0.39 | 0.66 | –0.10 | 0.06 | –0.02 |
| bio18 | 0.05 | –0.15 | –0.26 | 0.10 | –0.02 | –0.06 | 0.05 | 0.46 | –0.31 | 0.13 | –0.03 | 0.49 | 0.54 | 0.40 | 0.07 | 0.52 | 0.39 | 1.00 | –0.03 | –0.14 | –0.02 | 0.06 |
| bio19 | 0.46 | –0.11 | 0.47 | –0.53 | 0.17 | 0.53 | –0.49 | –0.49 | 0.62 | 0.20 | 0.53 | 0.84 | 0.68 | 0.62 | –0.24 | 0.74 | 0.66 | –0.03 | 1.00 | 0.03 | 0.07 | –0.04 |
| elev | –0.05 | 0.59 | 0.43 | –0.14 | 0.04 | –0.02 | 0.03 | –0.38 | 0.15 | –0.20 | 0.03 | –0.07 | –0.02 | –0.11 | 0.08 | –0.03 | –0.10 | –0.14 | 0.03 | 1.00 | –0.03 | –0.09 |
| gm_lc | 0.14 | 0.10 | 0.03 | 0.09 | 0.26 | 0.01 | 0.11 | 0.05 | 0.03 | 0.27 | 0.04 | 0.07 | 0.07 | 0.05 | 0.07 | 0.08 | 0.06 | –0.02 | 0.07 | –0.03 | 1.00 | –0.03 |
| gm_ve | –0.05 | –0.15 | –0.09 | –0.03 | –0.13 | 0.00 | –0.06 | 0.09 | –0.06 | –0.09 | –0.01 | –0.01 | 0.00 | –0.01 | –0.03 | –0.01 | –0.02 | 0.06 | –0.04 | –0.09 | –0.03 | 1.00 |

**Table S2.** Potential suitable area of *Melanophila acuminata* in China under different scenarios.

| Scenarios, times | Low suitable area  (km^2^) | Moderate suitable  area (km^2^) | High suitable  area (km^2^) |
| --- | --- | --- | --- |
| Current | 3.49 | 9.16 | 4.08 |
| SSP126, 2041–2060 | 3.18 | 8.97 | 4.13 |
| SSP245, 2041–2060 | 3.33 | 8.59 | 3.40 |
| SSP585, 2041–2060 | 3.28 | 7.86 | 3.07 |
| SSP126, 2061–2100 | 3.21 | 8.90 | 3.87 |
| SSP245, 2061–2100 | 3.29 | 8.43 | 3.50 |
| SSP585, 2061–2100 | 2.98 | 6.80 | 2.62 |

**Figure S1.** Pearson correlation analysis of environmental variables.

**Figure S2.** The ROC curve for predicting the potential distribution range of *Melanophila acuminata* based on the MaxEnt model.
